# Retraction: The role of microRNAs in the tumorigenesis of ovarian cancer

**DOI:** 10.3389/fonc.2020.00585

**Published:** 2020-04-07

**Authors:** Frontiers Editorial Office

The journal retracts the June 13, 2013 article cited above.

Following publication, the authors contacted the Editorial Office to request that their article be corrected because of the inappropriate manner in which the article was written, which specifically involved improper reuse of text from previous articles. An investigation was conducted in accordance with our established procedures that confirmed the extensive and dispersed nature of the overlap; therefore, the article has been retracted.

This retraction was approved by the Chief Editors of Frontiers in Oncology and the Editor-in-Chief of Frontiers. The authors did not agree to this retraction.

